# Periodontal probing of an impacted tooth recovered through a surgical-orthodontic approach: a case report

**DOI:** 10.1186/1752-1947-8-25

**Published:** 2014-01-27

**Authors:** Maria Teresa Dinoi, Mariano Lacarbonara, Salvatore DiMartino, Annalisa Monaco, Giuseppe Marzo

**Affiliations:** 1 Department MeSVA, School of Dentistry, University of L’Aquila, L’Aquila, Italy

**Keywords:** Impaction, Incisor, Orthodontics, Periodontium, Probing

## Abstract

**Introduction:**

The aim of this work was to assess the periodontal support of a central upper incisor recovered through a surgical-orthodontic approach compared to the spontaneously erupted contralateral incisor.

**Case presentation:**

This case study describes an 8-year-old Caucasian female with an impacted upper right central incisor. Surgical-orthodontic treatment was performed to reset the impacted dental element in the arch. Periodontal probing was performed of all sites (mesio-buccal, central-buccal, disto-buccal, mesio-palatal, central-palatal and disto-palatal) of the recovered impacted tooth and the contralateral tooth. The results were compared to determine whether the treated element showed signs of periodontal injury.

**Conclusions:**

Most of the probing results on both her right and left incisors gave values of approximately 3mm, which were not considered pathological. Both dental elements had adequate and physiological osseous attachments.

## Introduction

The eruption of permanent teeth in the arch is regulated by strict genetic control, which guides the correct formation of the various dental gems and their eruption in the arch in the expected position. Under certain anatomical conditions, trauma or infective processes involving the corresponding deciduous teeth may cause alterations of the eruptive process, preventing the tooth from appearing in the oral cavity within the physiological eruption time frame and in an ectopic position. A tooth is considered to be "impacted" when it has not appeared in the arch within the maximum time limit of its physiological eruption, its root apices are closed and, consequently, it lacks eruption ability. The incidence of dental impactions has been reported to vary between 5.6% and 18.8%, with a higher frequency in women [1]. The teeth that are most frequently impacted are the lower and upper third molars (20 to 30%), followed by the upper canines (85% with palatal dislocation), lower second premolars (0.3%), and central upper incisors (0.1%) [2-4].

Several classifications can be used to evaluate the degree of the impaction of the dental elements. These classifications are based on different factors, such as the degree of impaction (that is, total versus partial) [5], number of impacted teeth (that is, single versus multiple) [5], duration of impacted (that is, temporary versus permanent), and cause of impaction (that is, primitive versus secondary). In particular, primitive impaction is caused by intrinsic factors, such as the anatomy and tilt of the tooth, whereas secondary impaction is caused by external factors, such as cystic pathologies, supernumerary teeth, and neoformations [5]. The etiopathogenesis of dental impactions is vast. Causes of dental impaction can be divided into general, local, and structural. General causes include genetics, hypofunction or hyperfunction of the endocrine system, metabolic dysfunction, and infectious diseases [6]. Local causes may be connected to the deciduous tooth (ankylosis, premature loss, and periapical chronic phlogosis) or to the permanent tooth (radicular ankylosis, corono-radicular morphological alterations, and positional anomalies) [6]. Structural causes include maxillary hypoplasia, serious hyperdivergence, skeletal open-bite, and congenital pathologies of the maxillofacial system [6].

Several therapies are possible, including classic orthodontic treatment, combined surgical-orthodontic treatments, conservative surgery, and radical surgical treatment [6]. In the simplest cases of tooth retention, a classical orthodontic treatment should be chosen. When the impacted tooth shows location and inclination anomalies or a particular corono-radicular morphology, a combined surgical-orthodontic treatment should be chosen. When the tooth eruption is hampered by a pathological condition (a cyst or odontoma) and its position in the arch is conditioned by the removal of the obstacle, conservative surgical treatment should be selected. In the case of serious anomalies in the tooth anatomy or location, or at the patient’s request, radical surgical treatment (extraction) may be chosen. Recovering the teeth in the arch is important, to ensure that the patient will have adequate functionality and good aesthetics. It is especially important at a young age, to ensure trophism in adjacent tissues and to maintain space for aesthetic and functional reasons.

## Case presentation

Here, we describe the case of an 8-year-old Caucasian girl, in the mixed dentition period. An extraoral examination did not reveal facial asymmetry. Intraoral examination showed that her dental development was age-appropriate, except for the absence of her central upper right incisor. The probable cause of the lack of eruption of her incisor was connected to a traumatic event that occurred during her childhood. As reported in her anamnesis, she fell at approximately 2 years of age. This trauma caused the impaction of her deciduous upper central right incisor, producing a delay in the formation of the corresponding permanent tooth and subsequent impaction. No pathologies or situations that may cause eruption anomalies were noted from her family or medical history.

**Figure 1:**
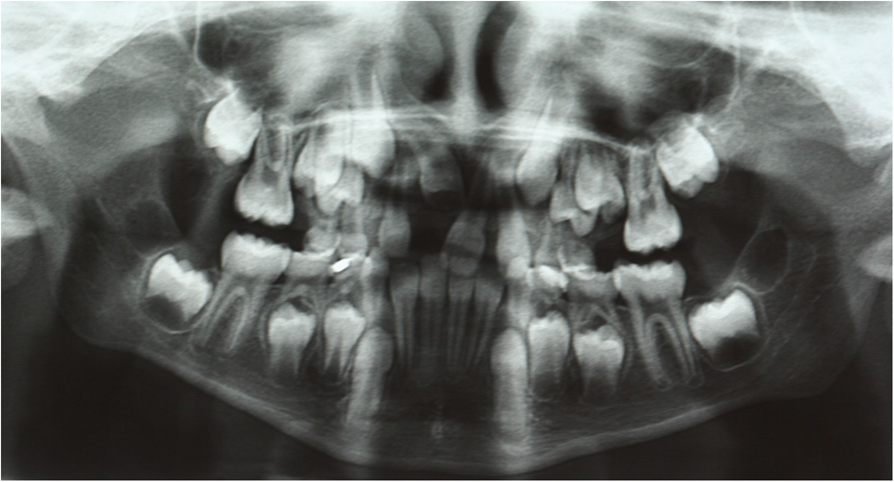
Initial panoramic radiography.

To determine an adequate treatment plan, a panoramic radiograph of her arches and a projection teleradiograph of her cranium in the latero-lateral position were needed for cephalometric evaluations. The panoramic radiograph confirmed the suspected diagnosis; her upper right central incisor was impacted, the incisor radicular apex was closed, and the tooth was unable to erupt (Figure 1). A combined surgical-orthodontic treatment was selected, which included surgical exposure in the proximity of the impacted incisor, traction of her impacted incisor into the dental arch using an anchorage device, and a period of functional orthodontics to improve the shape of her arches.

**Figure 2:**
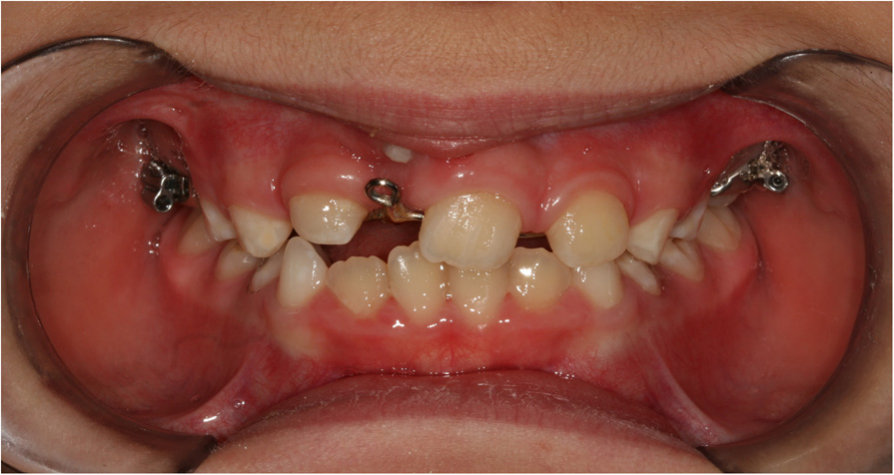
**Splint being cemented.** Intraoral photo.

**Figure 3:**
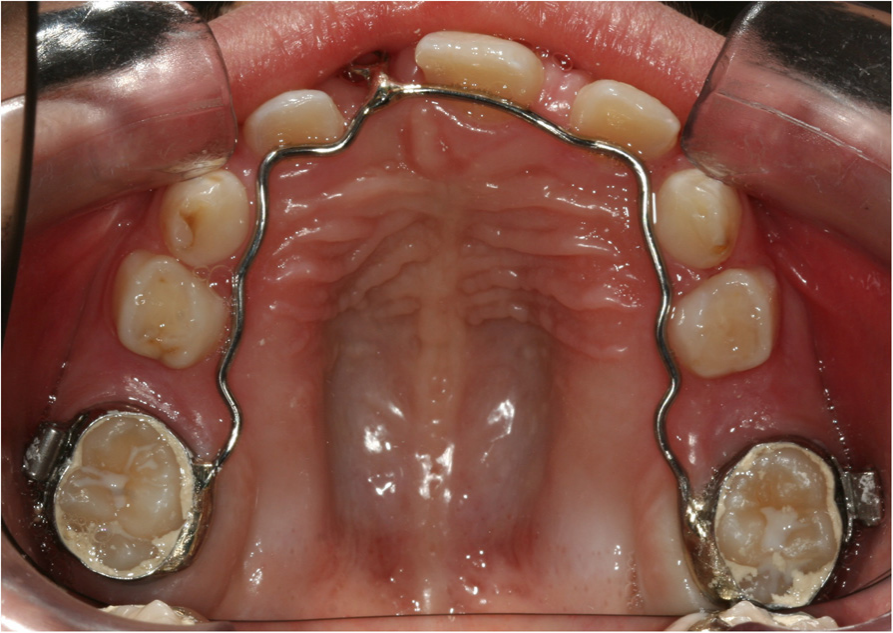
**Splint being cemented.** Occlusal intraoral photo.

**Figure 4:**
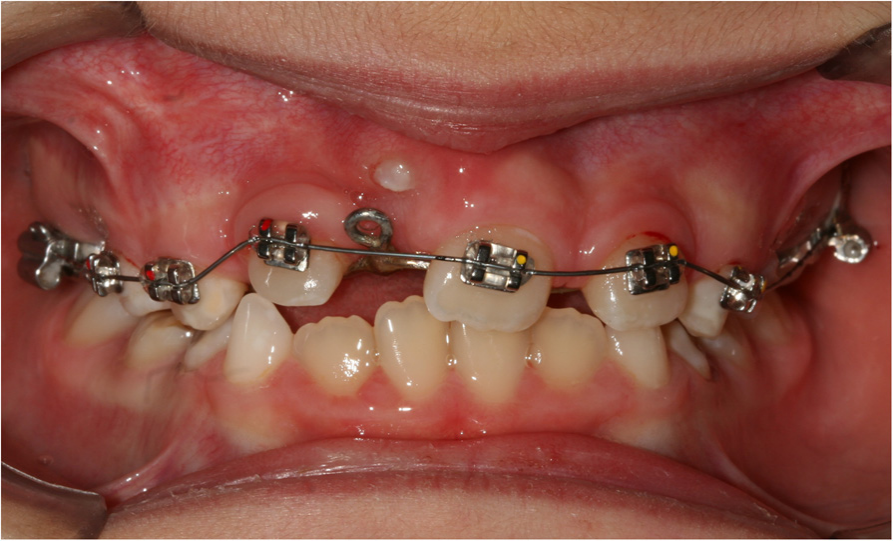
Banding of the upper arch.

**Figure 5:**
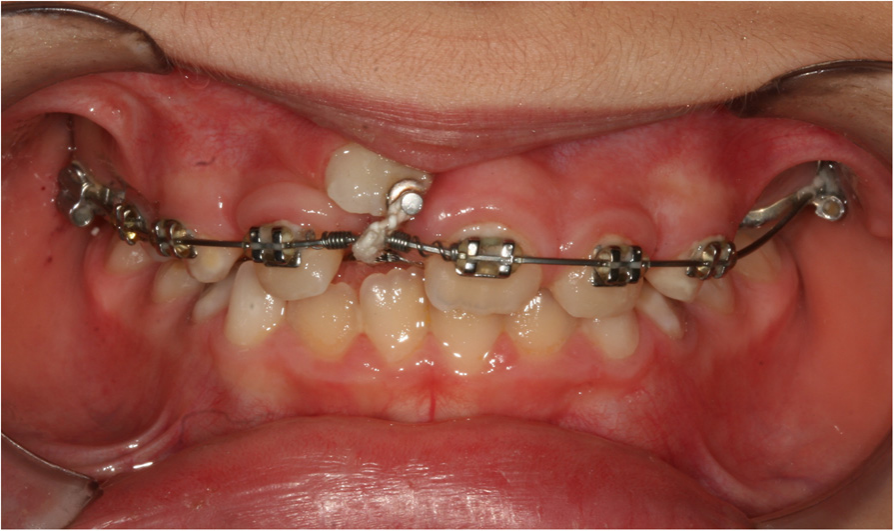
Expansion spring being positioned.

During the first session, an impression was taken with a band on her upper sixth to create a splint with an eyelet in zone 11, which was used later to apply traction to her impacted tooth. The splint was cemented (Figures 2 and 3). After 15 days, the upper arch was banded with pre-torque and pre-angled brackets. The first arch utilized was a 36mm (0.014-inch) nickel-titanium (Ni-Ti) round arch. Banding was performed using her deciduous teeth to ensure a better anchorage (Figure 4). Approximately 20 days after banding, surgical exposure was performed. A button was placed at her crown level and tied with an elastic wire to the eyelet of the auxiliary device to start traction, which was applied slowly, replacing the elastic wire approximately every 15 days. During the next session (15 days after surgical exposure), her stitches were removed, and the arch was replaced with a 0.016" × 0.022" rectangular Ni-Ti wire. In the same session, an expansion spring was fixed between her left central incisor and her lateral right incisor to make space for the settling of her central incisor (Figure 5). Approximately 60 days after exposure surgery, her tooth was visible in the arch. The contralateral tooth was correctly positioned at the start of treatment to allow a correct progression of her right incisor.

**Figure 6:**
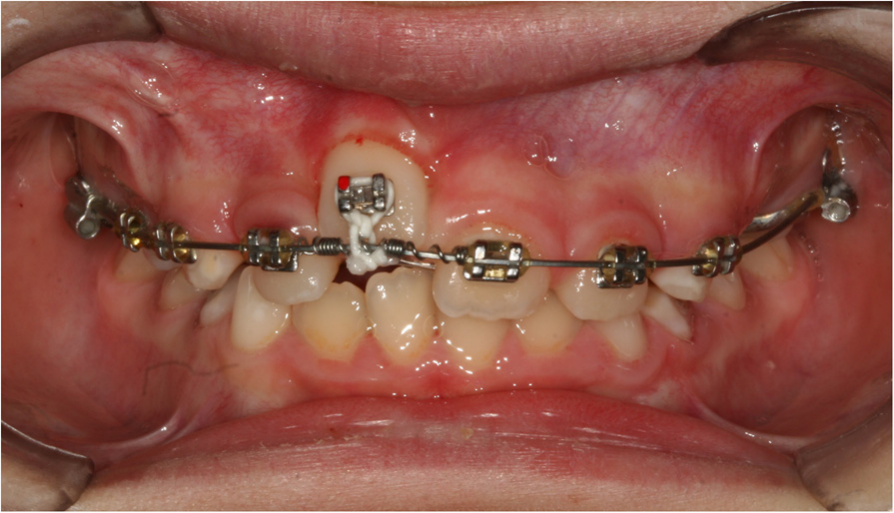
Tooth has almost reached the correct eruption zone.

**Figure 7:**
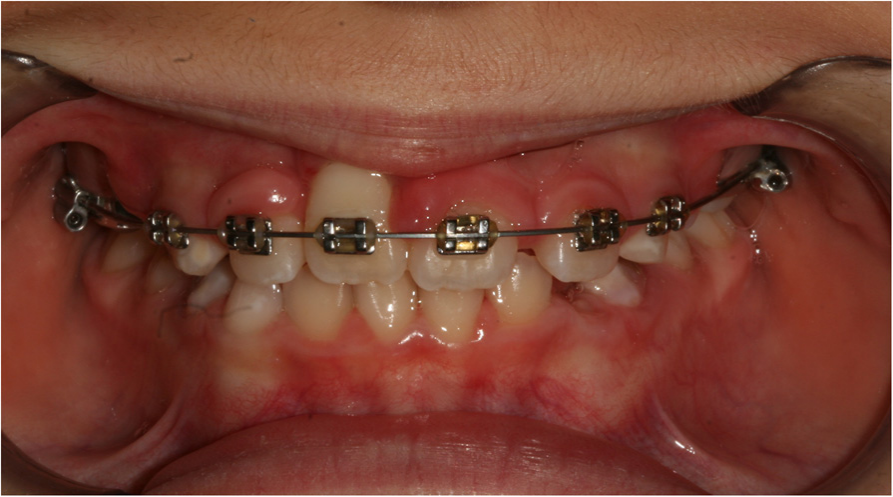
Tooth has reached its physiological position.

Once her incisor reached the proximity of the correct eruption position, the splint eyelet was removed, and the button was replaced with a bracket that was later directly tied to the arch (Figure 6). About 3 months after banding, the internal device was removed, and her tooth had reached its normal position (Figure 7). Once the established objectives were met, the bands were removed. The fixed treatment lasted a total of 7 months.

**Figure 8:**
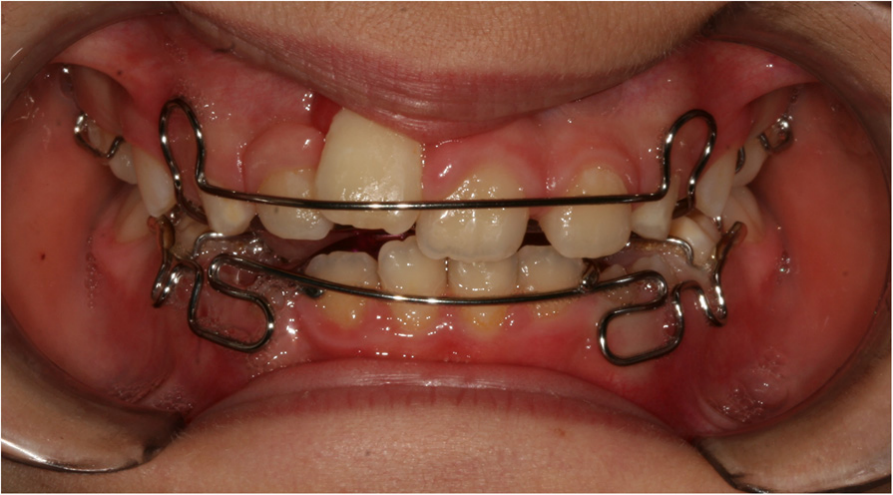
Functional orthodontics.

**Figure 9:**
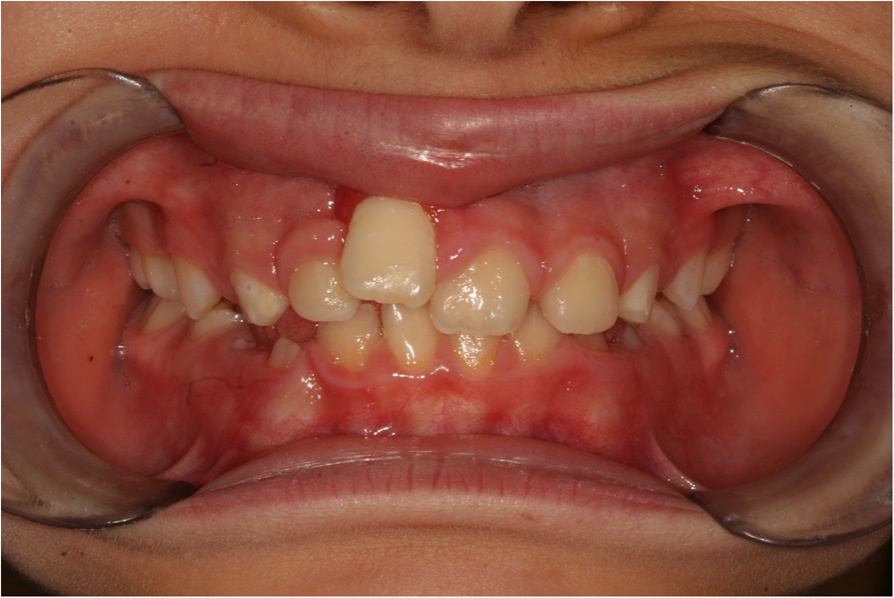
Final intraoral photo.

We continued with orthodontic treatment via two Schwarz’s plates, to obtain a slow expansion of the arches and improve their shape, postponing the final alignment of dental elements to a later stage when dental development was completed (Figure 8). After treatment, a defect was visible in her smile at the gingival attachment level of her right central incisor, which was clearly more apical when compared to the contralateral tooth (Figure 9).

To check if there had been a loss of tooth osseous support, periodontal probing of her two central upper incisors was performed. We compared the measurements to establish whether her right incisor had received any periodontal damage during traction. With a healthy periodontium, the distance between the gingival margin and the underlying bone should not exceed 3mm. When periodontal disease develops, there is a loss in the tooth bony support that partially or totally involves the root. The resulting "periodontal pocket", or deepening of the gingival sulcus, is defined as pathological when the loss of attachment exceeds 4 to 5mm. A graduated periodontal probe, with a 0.5mm-diameter rounded tip and a colored area extending from 3.5 to 5.5mm, was used for probing. All her teeth and all levels (mesio-buccal, center-buccal, disto-buccal, mesio-palatal, center-palatal, disto-palatal) were probed. The probe was gently inserted in the tooth-gingival sulcus, with a force of about 25 to 30*g*, held parallel to the apical surface, and moved along the tooth surface until resistance was encountered. The probing depth was read on the probe, using the height of the colored area with respect to the gingival margin as a reference [7-9].

The results of the probing are shown in Table 1. The probing results for her right incisor were very similar to those of her left incisor. The mesio-buccal, mesio-palatal, and center-palatal probing results were identical (3 and 4mm). The center-buccal and disto-buccal probing results of the recovered incisor were 1mm less than those of the contralateral probing. Finally, the disto-palatal probing result of the recovered tooth was 1mm greater than that of the contralateral tooth. We concluded that most of the probing values of her right and left incisors were around 3mm and were not indicated as pathological. Both dental elements had suitable and physiological osseous attachments.

**Table 1:** Results of periodontal probing after surgical-orthodontic treatment

	**Right incisor**	**Left incisor**
**Mesio-buccal**	3	3
**Center-buccal**	2	3
**Disto-buccal**	2	3
**Mesio-palatal**	3	3
**Center-palatal**	4	4
**Disto-palatal**	4	3

## Conclusions

The aim of this work was to assess the level of osseous attachment of an impacted tooth recovered through a surgical-orthodontic approach, compared to its spontaneously erupted contralateral counterpart. After recovering the included tooth through a fixed orthodontics treatment approach, periodontal probing of the recovered and contralateral incisors was performed to determine whether the treatment had caused serious osseous damage. The orthodontically recovered tooth had adequate periodontal support that was very similar to that of the contralateral tooth. The probing results were physiological and demonstrated that no loss of osseous support had occurred. This result was due to a good surgical exposure, which was achieved as conservatively as possible. The periodontal tissues were respected throughout the treatment. Finally, the orthodontic treatment used anchorages and light and constant forces, without damaging the tractioned tooth or the adjacent teeth.

## Consent

Written informed consent was obtained from the patient's guardian for publication of this case report and any accompanying images. A copy of the written consent is available for review by the Editor-in-Chief of this journal.

## Competing interests

The authors declare that they have no competing interests.

## Authors’ contributions

MTD conceived of the study, and participated in its design and coordination and helped to draft the manuscript. ML conceived of the study, and participated in its design and coordination and helped to draft the manuscript. SDM conceived of the study, and participated in its design and coordination and helped to draft the manuscript. AM conceived of the study, and participated in its design and coordination and helped to draft the manuscript. GM conceived of the study, and participated in its design and coordination and helped to draft the manuscript. MTD, ML and SDM were involved in the writing of the manuscript, GM had the idea for the manuscript, and AM reviewed the manuscript. All authors read and approved the final manuscript.
